# Automated detection of cognitive symptom severity and mild cognitive impairment in Parkinson’s disease

**DOI:** 10.1002/alz.085895

**Published:** 2025-01-09

**Authors:** Franco Javier Ferrante, Daniel Escobar Grisales, María Fernanda Lopez, Pamela Lopes da Cunha, Lucas Sterpin, Pedro Chaná Cuevas, Edinson Muñoz, Claudio Estienne, Eugenia Hesse, Lucía Amoruso, Juan Rafael Orozco Arroyave, Adolfo M. Garcia

**Affiliations:** ^1^ Cognitive Neuroscience Center, University of San Andrés, Victoria, Buenos Aires Argentina; ^2^ Consejo Nacional de Investigaciones Científicas y Técnicas (CONICET), Buenos Aires Argentina; ^3^ Universidad de Buenos Aires, Buenos Aires Argentina; ^4^ GITA Lab, Universidad de Antioquia, Medellín, Antioquia Colombia; ^5^ Hospital Nacional Posadas, El Palomar, Buenos Aires Argentina; ^6^ Universidad de Flores, Ciudad Autónoma de Buenos Aires, Buenos Aires Argentina; ^7^ Consejo Nacional de Investigaciones Científicas y Técnicas (CONICET), Ciudad Autónoma de Buenos Aires, Buenos Aires Argentina; ^8^ Cognitive Neuroscience Centre, University of San Andres, Victoria, Buenos Aires Argentina; ^9^ Centro de trastornos del movimiento (CETRAM), Lo Espejo, Región Metropolitana Chile; ^10^ Facultad de ciencias médicas, Universidad de Santiago de Chile, Estación Central, Santiago de Chile Chile; ^11^ Universidad Santiago de Chile, Estación Central, Santiago de Chile Chile; ^12^ Instituto de Ingeniería Biomédica, Ciudad Autónoma de Buenos Aires, Buenos Aires Argentina; ^13^ Universidad de San Andrés, Victoria, Buenos Aires Argentina; ^14^ Basque Center on Cognition, Brain and Language (BCBL), San Sebastián, Gipuzkoa Spain; ^15^ Ikerbasque, Basque Foundation for Science, Bilbao, Biscay Spain; ^16^ Pattern Recognition Lab, Friedrich‐Alexander Universität, Erlangen‐Nürnberg, Erlangen Germany; ^17^ Global Brain Health Institute, University of California, San Francisco, CA USA

## Abstract

**Background:**

Beyond dementia syndromes, cognitive symptoms are highly prevalent in Parkinson’s disease (PD), often manifesting as mild cognitive impairment (MCI). Yet, their detection and characterization remain suboptimal because standard approaches rely on subjective impressions derived from lengthy, univariate tests. Here we introduce a novel approach to detect cognitive symptom severity and identify MCI in PD using fully automated word property analyses on brief verbal fluency tasks.

**Methods:**

A total of 384 Spanish speakers with PD completed taxonomic, thematic, and phonemic fluency tasks, requiring them to name animals, supermarket items, and words starting with /p/, respectively. We automatically quantified six objective properties from each word: semantic variability (SV), granularity, length, frequency, phonological neighborhood, and concreteness. In Study 1, these properties were fed to a support vector regression with hyperparameter tuning to predict Mattis Dementia Rating Scale (MDRS) scores as indices of cognitive severity. Predicted MDRS scores were correlated with actual MDRS scores, adjusting for clinical factors (disease duration, motor symptom severity, medication dose). In Study 2, we used the same properties to compare (via a generalized linear model, adjusting for clinical variables) and classify (via ridge regression) between a carefully matched sample of 70 patients with and 75 patients without MCI. Feature importance analyses were conducted in both studies to identify which properties best captured patients’ cognitive profiles.

**Results:**

In Study 1, MDRS scores predicted by word properties were strongly correlated with actual MDRS scores (R = 0.53, p = .02). In Study 2, patients with MCI exhibited lower SV in taxonomic and thematic fluency, lower granularity and length in taxonomic fluency, higher phonological neighborhood in taxonomic fluency, and higher concreteness in phonemic fluency (p‐values < .05). Machine learning analysis of all properties showed robust subject‐level discrimination between patients with and without MCI (AUC = 0.79). Across both studies, machine learning results were mainly most consistently driven by SV and granularity, pointing to reduced semantic consistency and precision as key cognitive markers of PD.

**Conclusions:**

Automated word property analysis from brief fluency tasks can predict cognitive symptom severity and distinguish cognitive phenotypes of PD, opening new avenues for scalable neuropsychological screenings.

